# Collaborative Emission Reduction Model Based on Multi-Objective Optimization for Greenhouse Gases and Air Pollutants

**DOI:** 10.1371/journal.pone.0152057

**Published:** 2016-03-24

**Authors:** Qing-chun Meng, Xiao-xia Rong, Yi-min Zhang, Xiao-le Wan, Yuan-yuan Liu, Yu-zhi Wang

**Affiliations:** 1 School of Management, Shandong University, Jinan, Shandong 250100, China; 2 School of Mathematics, Shandong University, Jinan, Shandong 250100, China; 3 China Bohai Bank Co., Ltd., Tianjin, 300204, China; 4 Institute of Ecology and Biodiversity, School of Life Sciences, Shandong University, Jinan, Shandong 250100, China; Nankai University, CHINA

## Abstract

CO_2_ emission influences not only global climate change but also international economic and political situations. Thus, reducing the emission of CO_2_, a major greenhouse gas, has become a major issue in China and around the world as regards preserving the environmental ecology. Energy consumption from coal, oil, and natural gas is primarily responsible for the production of greenhouse gases and air pollutants such as SO_2_ and NO_X_, which are the main air pollutants in China. In this study, a mathematical multi-objective optimization method was adopted to analyze the collaborative emission reduction of three kinds of gases on the basis of their common restraints in different ways of energy consumption to develop an economic, clean, and efficient scheme for energy distribution. The first part introduces the background research, the collaborative emission reduction for three kinds of gases, the multi-objective optimization, the main mathematical modeling, and the optimization method. The second part discusses the four mathematical tools utilized in this study, which include the Granger causality test to analyze the causality between air quality and pollutant emission, a function analysis to determine the quantitative relation between energy consumption and pollutant emission, a multi-objective optimization to set up the collaborative optimization model that considers energy consumption, and an optimality condition analysis for the multi-objective optimization model to design the optimal-pole algorithm and obtain an efficient collaborative reduction scheme. In the empirical analysis, the data of pollutant emission and final consumption of energies of Tianjin in 1996–2012 was employed to verify the effectiveness of the model and analyze the efficient solution and the corresponding dominant set. In the last part, several suggestions for collaborative reduction are recommended and the drawn conclusions are stated.

## Introduction

### Background

In most cities, air quality is mainly influenced by the concentration of air pollutants. As a major industrial power, China relies on secondary industries that bring about rapid economic development. However, given that these industries have large energy consumption, they also emit massive amounts of air pollutants. Emissions from fossil energy combustion, when not properly diffused in time, can seriously affect air quality. China has promulgated an air pollution index (API) and an air quality index (AQI) for assessing air quality, and the calculations of these indexes revealed that SO_2_ and NO_X_ are the key factors affecting air quality.

Since the "Kyoto Protocol" was signed, China has been focused on reducing the emission of greenhouse gases, particularly CO_2_. In recent years, PM2.5 has aroused much attention, and its harmful contents mainly originate from energy combustion, power generation, metallurgy, petroleum and chemical engineering, as well as other human activities. According to the recent findings of the Chinese Academy of Sciences (CAS), even if the sources of coal combustion, industrial pollution, and secondary inorganic aerosols are merged and computed, the emission from fossil fuel combustion would still be the main source of PM2.5 pollution in Beijing. Moreover, PM2.5 can be converted by oxides of sulfur and nitrogen. In the developing world, coal combustion is the primary means for home heating and energy supply.

Fossil fuel combustion is main cause of severe environmental problems that hinder the promotion of the social development and living quality of people. Although the public has been aware of the seriousness of the implications of such problems and have implemented effective attempts to address them, such as improving combustion technologies, developing new energies, changing lifestyles, adjusting industrial structure, etc., fossil fuels remain as the main form of energy that supports the social development in China. Fossil fuels can be categorized into various kinds and be used in numerous ways. The amounts of pollutants generated from different kinds of energies in different ways vary but are associated to a certain extent. Therefore, this research tries to explore an energy use scheme that realizes the minimum pollution emissions while guarantying energy use in social development and people's life.

### Problems in collaborative reduction

SO_2_ in air is mainly produced from combustion of coal, oil, and other sulfur-containing fuels, and more than 90% of the SO_2_ emissions in China are sourced from coal [1]. Since the 1980s, China has started stressing SO_2_ control by adopting various technical measures, including adjusting coal structure, improving clean coal utilization, using clean combustion technology, and encouraging key emission departments to introduce fuel gas desulfurization facilities. Moreover, several economic management measures, including decommissioning small thermal units, formulating emission standards for coal-fired power plant, and making total amount control, have been adopted to further control the amount of SO_2_ emissions.

NO_X_ plays an important role in the formation of photo-chemical smog, and is the main driver in turning acid rain of the nitric acid type into the sulfuric acid type. Given that China has had a late start in NO_X_ control, NO_2_ emissions grew rapidly during the period of “11th five-year plan”. In the “12th five-year plan”, NO_X_ became the second air pollutant that required total quantity control in addition to SO_2_. At present, China is adopting various measures for the prevention and control of NO_X_, such as low NO_X_ combustion technology, smoke denitrification technology, capacity control. In addition, the collaborative reduction of SO_2_, NO_X_, and the precursors of PM2.5 exerts a significant improvement to the hazy weather and the PM2.5 prevention and control.

CO_2_ is recognized worldwide as the main greenhouse gas. Ever since global warming has aroused wide interest, reducing greenhouse gas emissions has become an important target of governments around the world. The Intergovernmental Panel on Climate Change (IPCC) indicated in their fourth assessment report that CO_2_ is the most significant anthropogenic greenhouse gas, and its concentration increase is mainly caused by the use of fossil fuels [2]. According to greenhouse gas reduction target released in China, by 2020, CO_2_ emission per GDP will be 40%–50% lower than that in 2005. China has a relatively late start in carbon reduction than prevention and control of SO_2_, NO_X_, and other air pollutants, and it has readjusted economic structures, formulated laws and regulations, and disseminated social education propaganda since the "Kyoto Protocol" was signed.

Greenhouse gases oriented by CO_2_ and air pollutants oriented by SO_2_ and NO_X_ are all sourced from combustion of fossil fuels, in a medium (air) and are consistent in reduction measures (adjusting industrial structure and limiting production capacity, etc.). Therefore, to reduce emissions of both polluting gases and greenhouse gases and formulate reasonable energy technologies based on these features and on economic and management methods, policies, and regulations, the collaborative reduction way is developed.

In consideration of the overall scientific and technical level and current national conditions in China, fossil fuels cannot be fully replaced by any other energies, thus will still be dominating for a period of time in the future. The main fossil fuels currently used in China can be divided into three types: coal, oil, and natural gas. Although they all produce three kinds of polluting gases stated above, the quantities of emissions differ greatly in generating the same amount of energy because of the differences in combustion mechanisms. For example, coal contains more sulfur and produces much more SO_2_ than oil and gas, whereas oil produces much more NO_X_ than the other two kinds of fossil fuels. Natural gas, which is considered a clean source of energy, produces much more CO_2_ than the other two kinds of energies. Aside from the differences in energy types, different ways of usage can also affect the emission proportions of these three kinds of gases. For example, industrial sectors with more advanced technologies for fuel gas desulfurization and denitrification produce less SO_2_ in coal combustion than other sectors; however, since they produce more NO_X_ because of their use of high temperatures.

In consideration of the differences in energy types and combustion ways, the collaborative reduction method proposed in this research mainly discusses about reasonable arrangement of different energies in different sectors and maximum consumption reduction while guaranteeing production, so as to realize unification of ecological, economic and social benefits.

At present, collaborative reduction has aroused common attention of both local and international scholars. Bollenet al., (2009) analyzed the efficiency of collaborative governance of air pollution and global warming in view of economics [3]. Rafaj et al., (2011) compared the differences of SO_2_, NO_X_, and other emissions before and after the release of the agreement on green gas control [4], and showed that the policies have effectively reduced the emissions of air pollutants. Mao et al., (2012) studied economic ways and corresponding evaluations of collaborative reduction in China's electric power industry and steel industry [5–6], while Qin (2012) studied the benefits of collaborative reduction in Shenzhen City [7]. The above researchers analyzed the economic benefits of collaborative reduction from the perspective of economics, and explored ways of implementing collaborative reduction in specific industries from a technical level. However, none of them studied about reasonable distribution of energies based on pollutant production features of different energies and corresponding reduction benefits. In this research, the collaborative reduction of SO_2_, NO_X_, and CO_2_ is explored by considering their common features. This research, through mathematical modeling and systematical analysis, strives to provide the optimal scheme for regional collaborative emission reduction of greenhouse gases and air pollutants in order to promote sustainable development of ecological system.

### Introduction of the multi-objective optimization

Multi-objective optimization, which is used to achieve the optimal solutions concurrently with multiple objectives considered in a certain space of decision, was first proposed by economist Pareto in 1927. Multiple objectives are often incommensurable because of the absence of unified criteria or measurement units among objectives and contradictory because of the impossibility of finding a solution to make multiple objectives achieve the optimum in most cases [8]. In 1951, Koopmans put forward the concept of Pareto efficient solution in multi-objective optimization [9]. In the same year, Kuhn and Tucker studied the necessary and sufficient conditions for achieving the optimal solutions in general multi-objective optimization [10]. In 1968, Johnsen published the first monograph about multi-objective optimization [11], and during the 1970s–1980s, basic theories of multi-objective optimization were established with the efforts of many scholars.

Based on the theory of multi-objective planning, the preference of decision makers to the objectives is generally required to evaluate the advantages and disadvantages of a solution. In 1979, Hwang and Masud [12] proposed to classify the solving methods for multi-objective problem into beforehand evaluation, intermediate evaluation, and afterward evaluation according to expressions of the preference information. In the recent years, intermediate evaluation and afterward evaluation have become increasingly popular, and more and more intelligent algorithms have been introduced to solve the multi-objective programming with the development of artificial intelligence algorithm. Moreover, multi-objective optimization has increasingly been applied in chemical production, material processing, logistic design, and other social problems [13,14]. Flegiel et al., [15] presented two slightly different cumene process designs and used multi-objective optimization to examine two trade-offs, i.e., total capital cost (TCC) versus material loss and TCC versus utility cost. Sebnem et al., [16] developed a decision support system for the management of anaerobic digestion based biomass to energy supply chains in a cost effective and environment friendly manner. A fuzzy multi-objective mixed integer linear programming model was constructed as the most important mathematical method. Luquea [17] expanded the definition of multi-objective optimization problem by analyzing a new set of ‘‘equivalent reference points”, which allows generating the same Pareto optimal solution in reference point-based approaches. In view of the linear multi-objective optimization, Xu et al., [18] proposed the concept of predominant set corresponding to efficient solutions in multi-objective programming in his research on removable pole theorem of linear multi-objective programming.

## Methods of Mathematical Modeling

Four types of mathematical methods are introduced in this section, namely the Granger causality test to analyze the causality between air quality and pollutant emission, a function analysis to determine the quantitative relation between energy consumption and pollutant emission, a multi-objective optimization to set up the collaborative optimization model considering energy consumption, and an optimality condition analysis using the mathematical features of the multi-objective optimization model to design the optimal-pole algorithm and get an efficient collaborative scheme of reduction.

### Granger causality test model

Granger causality test model is an econometric analysis tool put forward by Clive Granger, a Nobel laureate in economics [19]. According to this theory, if the results of predicting variable Y based on previous information containing *X* and *Y* is better than that based on previous information containing *Y*, i.e., variable *X* is conducive to explaining future change of *Y*, *X* is considered to be the Granger reason of *Y*. In the Granger test, establishing the model in Eq 2.1 is the main process, where *u*_*t*_ is the white noise sequence; *α*,*β* are coefficients of the regression model; and *p*,*q* are the lag orders.

$$\left\{ \begin{array}{l} Y_{t}=\alpha +\sum_{i=1}^{p}\alpha_{i}X_{t-i}+\sum_{j=1}^{q}\beta_{j}Y_{t-j}+u_{t}\\ Y_{t}=\alpha +\sum_{j=1}^{q}\beta_{j}Y_{t-1}+u_{t} \end{array}\right.$$ (2.1)

In this research, the econometric software Eviews7 is used in the Granger causality test to verify the causality between the quality of pollutant emissions and air quality.

### Functional relation between energy consumption and polluting gas emission

In this paper, energy refers to the fossil fuels that discharge CO_2_ and a variety of air pollutants including SO_2_, NO_X_, smoke, CO, and hydrocarbons after consumption.

#### Functional relation between fossil fuel combustion and SO_2_ emission

In China, coal contains around 0.5–3% of sulfur and oil contains 0.06–0.8%. In normal combustion conditions, sulfur in fuel oxidize into SO_2_. According to "The Methods for Predicting Quantity of Pollution Emissions "in" Resources (energy)—Environment—Economic Prediction and Research Report of The National 12th Five-Year Plan" (hereinafter referred to as "the Report"), the whole society is divided into several sectors with energy consumption, and the functional relation between energy consumption and quantity of SO_2_ emissions is obtained, as shown in Eq 2.2, where *x*_*ij*_ refers to the consumption quantity of the *j*th energy by the *i*th sector, *α*_*j*_ refers to the sulfur content of the *j*th energy, *β*_*j*_ refers to the sulfur conversion rate of the *j*th energy, and *γ*_*i*_ is the removal rate of the *i*th sector in energy consumption, i.e., desulphurization efficiency.

$$E_{so_{2}}=\sum_{i}\sum_{j}2\alpha_{j}\beta_{j}(1-\gamma_{1i})x_{ij}$$ (2.2)

#### Functional relation between fossil fuel combustion and NO_X_ emission

As oxysulfide is only sourced from sulfur in fuels, different from SO_2_, NO_X_ is influenced by more factors in formation. According to the Report, nitrogen molecules in oxidizing air are oxidized in high temperature, and nitrides in fuels are partially oxidized in the process of burning, becoming the main source of NO_X_. The functional relation of NO_X_ emissions in energy consumption is shown in Eq 2.3, where *x*_*ij*_ is as defined above, *η*_*ij*_ refers to the corresponding NO_X_ emission factor of the *i* sector in consuming the *j*th energy, and *γ*_2*i*_ is the removal rate of the *i*th sector in energy consumption, i.e., denitration efficiency.

$$E_{NO_{x}}=\sum_{i}\sum_{j}\eta_{ij}(1-\gamma_{2i})x_{ij}$$ (2.3)

In mathematical view, Eqs 2.2 and 2.3 can be converted into logarithmic form (Eq 2.4), wherein *E* denotes the total amount of pollutant emissions, *X* the total amount of energy consumption, *γ* is the pollutant removal rate, *α* is the constant term, and *ε* is the random error.

lnE=α+lnX+ln(1−γ)+ε (2.4)

#### Functional relation between fossil fuel combustion and CO_2_ emission

In combustion, fossil fuel converts its energy into heat energy mainly through carbon oxidation, and discharges large amount of CO_2_. Based on the calculation method proposed in the Report, the functional relation is concluded, as shown in Eq 2.5, where *x*_*ij*_ is as previously defined, *c*_*i*_ is the carbon emissions factor of the *j*th energy given by IPCC, and *ω*_*i*_ is the combustion loss rate of the *j*th energy.

$$E_{co_{2}}=\sum_{i}\sum_{j}0.98c_{j}(1-\omega_{i})x_{ij}$$ (2.5)

Based on these formulas, although SO_2_, NO_X_, and CO_2_ follow different mechanisms in generation, they are all determined by the total amount of different energies consumed and form a positive linear relation with energy consumption.

### Multi-objective optimization model for collaborative reduction

By analyzing the amount of emission of the three kinds of gases, the collaborative reduction in different ways of energy consumption is discussed in this subsection. The whole society is divided into *M* sectors, for which *N* types of energies are available. The decision variable *x* = (*x*_*ij*_), *i* = 1,⋯,*M*, *j* = 1,⋯*N* denotes the amount of the *j*th energy consumed by the *i*th sector, and *E*_*i*_(*x*), *i* = 1,⋯3 denotes the amount of SO_2_, NO_X_, and CO_2_ consumption, thereby, in the objective function, minimum values should be used, as shown below,

$$\min E(x)=(E_{1}(x),E_{2}(x),E_{3}(x))$$

The consumption variable needs to meet the following requirements. First, the energy supply should meet necessary requirements for social development. Given that *ρ*_*j*_, *j* = 1,2,⋯,*N* denotes the coefficient of converting the *j*th energy into standard energy, *D*_*i*_, *i* = 1,⋯,*M* and denotes the total amount of energy demanded by the *i*th sector in development, thereby, the energy supply condition is expressed as follows:

$$\sum_{j}\rho_{j}x_{ij}\geq D_{i},i=1,2,\ldots ,M$$ (2.6)

On the other hand, fossil fuels are exhaustible, and the possibility of energy exploitation is also very limited in today's society. Hence, the limitation of energy should be considered in the model such that if *S*_*j*_, *j* = 1,…,*N* denotes the upper limit of supply of the *j*th energy, the energy limiting conditions are shown as follows:

$$\sum_{j}x_{ij}\leq S_{j},i=1,2,\ldots ,N$$ (2.7)

Moreover, the consumption should be non-negative, i.e.,. *x*_*ij*_ ≥ 0, *i* = 1,2,…,*M*, *j* = 1,2,…,*N*.

To sum up, the obtained model for collaborative reduction is a nonlinear multi-objective programming with linear constraints, expressed as follows:

$$\begin{array}{l} \min E(x)=(E_{1}(x),E_{2}(x),E_{3}(x))\\ s.t.\left\{ \begin{array}{l} \sum_{j}\rho_{j}x_{ij}\geq D_{i},i=1,2,K,M\\ \sum_{i}x_{ij}\leq S_{j},j=1,2,K,N\\ x_{ij}\geq 0 \end{array}\right. \end{array}$$ (2.8)

### Analysis of optimality conditions of the model

To facilitate the analysis of the mathematical properties of the model, model 2.8 is converted into the standard form.

Given that *ρ* = (*ρ*_1_,*ρ*_2_,⋯,*ρ*_*N*_), *E*_*i*_ is an *N* × *M* matrix (elements in the ith line are 1 and others are 0), *b* = (*D*_1_,⋯,*D*_*N*_,−*S*_1_,⋯,−*S*_*M*_), and the slack variable is denoted as $x^{d}=(x_{1}^{d},\cdots ,x_{M+N}^{d})$, the constraint matrix is expressed as follows:

$$A_{(M+N)\times MN}=\left(\begin{array}{c} \begin{array}{l} A_{1}\\ \vdots \end{array}\\ A_{p}\\ \vdots \\ A_{M+N} \end{array}\right)=\left(\begin{array}{l} \begin{array}{ccc} -\rho & \cdots & 0\\ \vdots & \ddots & 0\\ 0 & \cdots & -\rho \end{array}\\ \begin{array}{ccc} E_{1} & \cdots & E_{N} \end{array} \end{array}\right)$$

Model 2.8 is equivalent to the programming of the following standard form:

$$\begin{array}{l} \min\;\text{C}(x)=(C_{1}^{T}x,C_{2}^{T}x,C_{3}^{T}x)\\ s.t.\left\{ \begin{array}{l} Ax-b+x_{}^{d}=0\\ x_{ij}\geq 0,x_{p}^{d}\geq 0,\;i=1,\cdots ,M,j=1,\cdots ,N,p=1,\cdots ,M+N \end{array}\right. \end{array}$$ (2.9)

In the following subsections, the mathematical properties of model 2.9 is discussed. The necessary and sufficient conditions for Pareto efficient solution are analyzed, and then the concept of dominant set of solutions is introduced to design the method of calculating the efficient solution that contains the dominant set.

#### Optimality conditions of the model

First, the necessary conditions for Pareto efficient solution of model 2.9 are given according to the relevant theory in [20].

**Theorem 1** If $\bar{x}$ is a weak Pareto efficient solution of model 2.9, $\lambda =(\lambda_{1},\lambda_{2},\lambda_{3})\in R_{+}^{3}$, $u=(u_{1}^{d},L,u_{MN}^{d})\in R_{+}^{MN}$, and $v=(v_{1}^{d},L,v_{MN}^{d})\in R_{+}^{M+N}$ meet the following three conditions, where in *C*(*C*_1_,*C*_2_,*C*_3_):

(λ,u,v)≠0 (2.10)

$$\lambda C^{T}-u+vA=0$$ (2.11)

$$u_{ij}x_{ij}=0,v_{p}x_{p}^{d}=0,i=1,\cdots ,M,j=1,\cdots ,N,p=1,\cdots ,M+N$$ (2.12)

Based on these equations, the sufficient conditions for Pareto efficient solution of model 2.9 are analyzed.

**Theorem 2** If $\bar{x}\in S$ (*s* denotes feasible region), $\lambda \in R_{+}^{3}\backslash \{0\},u\in R_{+}^{MN+M+N}$ and $v\in R_{+}^{M+N}$, given that Eqs 2.10–2.12 hold, then $\bar{x}$ must be a weak Pareto efficient solution of model 2.9. Furthermore, if *λ* > 0, then $\bar{x}$ must be a Pareto efficient solution.

#### Analysis of dominant set of efficient solutions of the model

By comparing the optimality conditions of model 2.9 and the K-T conditions of a single-objective constraint programming, the optimality conditions of their Pareto efficient solutions may be regarded as employing a non-negative weight to multi-objectives (*λ* in the theorem) to convert into the optimality conditions of solutions of the single-objective programming. Different weights can be regarded as preferences of the decision makers to different objectives, and different preferences correspond to different solutions. However, a multi-objective problem may contain an infinite amount of weight information which may correspond to multiple feasible solutions, hence, it is impossible to get all Pareto efficient solutions of a multi-objective problem. To solve this problem, the concept of dominant set is introduced.

For linear multi-objective programming in model 2.13 and weighted form *L*_*λ*_ in model 2.14, we derive the following expressions:

$$\begin{array}{l} \min\;\text{C}(x)={\left(C_{1}^{T}x,\cdots ,C_{p}^{T}x\right)}^{T}\\ s.t.\left\{ \begin{array}{l} A'x=b\\ x\geq 0 \end{array}\right. \end{array}$$ (2.13)

$$\begin{array}{l} \min\;\text{C}(x)=\sum_{k=1}^{p}\lambda_{k}C_{k}^{T}x\\ s.t.\left\{ \begin{array}{l} A'x=b\\ x\geq 0 \end{array}\right. \end{array}$$ (2.14)

Efficient solutions of the former and optimum solutions of the latter are highly consistent. Literature [18] concluded the following:

**Conclusion 1** Any $\lambda \in R_{++}^{p}$ has optimum solution, and $\bar{x}$ is an effective solution to model 2.13.

**Conclusion 2** If $\bar{x}$ is any efficient solution of model 2.13 $\lambda \in R_{++}^{p}$ and, the $\bar{x}$ is the optimum solution of *L*_*λ*_.

Considering the substantial connection between them [18], concluded the superior setto analyze parameter information in objectives corresponding to the efficient solutions.

**Definition 1** If $\bar{x}$ is an effective solution of model 2.13, the $\bar{E}=\{\bar{\lambda }\in R_{+}^{p},\bar{x}\;\text{is the optimal solution of}\;L_{\lambda }\}$ is the dominant set of $\bar{x}$

**Conclusion 3** If $\bar{x}$ is an effective solution of model 2.13 and its dominant set is

$$\bar{E}=\{\bar{\lambda }|\bar{\lambda }\in R_{++}^{p},\bar{\lambda }C^{T}\bar{x}\leq \bar{\lambda }C^{T}x^{\left(i\right)}\},$$

where *x*^(*i*)^ ∈ S*, and S* = {*x*^(1)^,…,*x*^(*r*)^} is the set of all poles of *S*.

For multi-objective programming, there are often infinite amount of efficient solutions, hence, the dominant set can be used to select the representing efficient solutions.

**Definition 2** If *x*_1_ and *x*_2_ represent two different efficient solutions, *E*_1_ and *E*_2_ represent dominant sets of model 2.13, and *E*_1_ ⊆ *E*_2_, the dominance of *x*_1_ is contained in *x*_2_, or *x*_1_ is removable relative to *x*_2_.

**Conclusion 4** (Theorem of removable) If $\bar{x}$ is a non-pole effective solution to model 2.13, then $\bar{x}$ is removable relative to a non-inferior pole of model 2.13.

#### Efficient solutions of the model and dominant set algorithm

By combining the optimality conditions of the multi-objective programming and the dominant set theory, this section presents an optimization algorithm to solve non-removable efficient solutions of the optimization model for energy saving and emission reduction and its corresponding dominant set.

#### Optimization algorithm

Step0 Solve all poles of feasible set of the model; set *S* = {*x*^*i*^, *i* = 1,2,…,*r*}, initial effective pole set S* = ∅ and *k* = 1.

Step1 For all *i*, substitute $\bar{x}=x^{i}$ into model 2.14 and verify if Eqs 2.10–2.12 hold. If so, *x*^*i*^ is an efficient pole, and S* = S* ∪ {*x*^*i*^}, *k* = *k*+1.

Step2 If *k* = *r*, all efficient points have been found and their set is recorded as S*, and is the set of target values of efficient poles in S*.

Step3 Solve the set of inequalities E^*j*^ = {*λ* | *λ*(*y*^*j*^ − y*) < = 0, ∀y* ∈ Y*}, where *y*^*i*^ is the target value of *x*^*i*^, and all non-removable poles and corresponding dominant sets are obtained.

## Empirical Analysis of Collaborative Reduction and the Emission—Investment Model in Tianjin

Tianjin City, an old and the largest coastal open city of China, has a continuous augment of energy consumption caused by rapid development of its industry-based economy. China’s Ministry of Environmental Protection once pointed out that unorganized emission phenomenon was serious in some industrial enterprises of Tianjin. The reason for selecting Tianjin for this research is because the energy consumption pattern in the city, which mainly consists of fossil fuels like coal, petroleum, and natural gas, is the typical pattern in most industrial cities, hence, Tianjin can be a representative in the empirical analysis of collaborative emission. We use data for Tianjin from 1996 to 2012 to verify the model. Because of the hysteretic nature of statistical yearbook issuing, the data from 2012 is the latest data acquired when we implemented this research.

### Demonstration of Granger causality test

**Fig 1:**
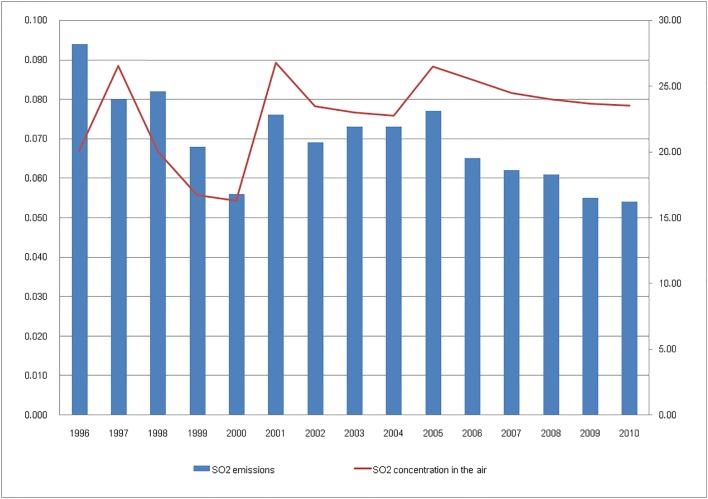
Trend diagram of SO_2_ emissions and its concentration in the air in Tianjin

Fig 1 shows the trend chart of SO_2_ emissions (ten thousand tons) and average SO_2_ concentration in the air (mg/m^3^) from 1996–2010 in Tianjin. Broken line represents the total amount of emissions over the years, and columnar data represent the average SO_2_ concentrations in the air. As seen from the figure, the SO_2_ content in the air is related to the emission of relevant pollutants. To get more accurate causality between them, Granger causality test is conducted to verify the relationship between the pollutant emissions and air quality.

Econometric software Eviews7 was used in the Granger causality test, and the results are shown below.

As is shown Table 1, when the "lags" is 2, and the null hypothesis "SO_2_DISCHARGED does not Granger Cause SO_2_ INAIR" is rejected, the probability of error 1 is up to 0.5209, and when the null hypothesis "SO_2_INAIR does not Granger Cause SO_2_DISCHARGED" is rejected, the probability of error 1 is 0.1467. Therefore, SO_2_ emission is the Granger cause affecting SO_2_ concentration in the air.

**Table 1:** Results of Granger Test.

Null Hypothesis	Obs	F-Statistic	Prob.
SO_2_DISCHARGED does not Granger Cause SO_2_INAIR	13	0.70827	0.5209
SO_2_INAIR does not Granger Cause SO_2_DISCHARGED		2.46362	0.1467

Pairwise Granger Causality Tests

Date: 01/0814 Time: 14:56

Sample: 1996 2010

Lags: 2

### Analysis of actual parameters among energy consumption, polluting gas emission

#### Analysis of control variables in the optimization model 2.8

Based on the specific data of Tianjin, the general classification in the Report, and statistical yearbook, as well as the different pollutant discharge coefficients in energy consumption, the society is divided into ten sectors, namely, agriculture, power generation, heat supply, oil refining, gas generation, industrial, construction, transportation, business, and life; fossil energies are classified into coal, coke, crude oil, gasoline, kerosene, diesel oil, fuel oil, natural gas, and gas. Thus, the model has 90 variables of corresponding standard form, resulting in a huge amount of calculation. For this reason, we merged the variables and made appropriate simplifications. According to the actual consumption of different energies by different sectors in Tianjin, in this paper, the city is divided into five sectors, namely, large-scale agriculture (farming, forestry, animal husbandry, fishery, and water conservancy), industry (power generation and heat supply), transportation, business, and life; and according to the similarity of pollution discharging coefficients in energy consumption by these sectors, the nine kinds of energies stated above are summarized as coal, oil, and natural gas.

#### Target parameters in the optimization model 2.8

According to the Report and statistical yearbook, the pollution discharging coefficients of all kinds of energies are sorted out, as shown in Table 2.

**Table 2:** Pollution Discharging Coefficients.

Product No.	Sectors	Energies	SO_2_ emission factor	NO_X_ emission factor	CO_2_ emission factor
			10^-3^kg/kg(m^3^)	10^-3^kg/kg(m^3^)	10^-3^kg/kg(m^3^)
1	Large-scale agriculture	Coal	12	3.75	1977.90
		Oil	18	8.26	2984.75
		Natural gas	0	1.462	2184.03
2	Industry	Coal	8.4	8	1977.90
		Oil	12.6	8.86	2984.75
		Natural gas	0	2.085	2184.03
3	Transportation	Coal	12	7.5	1977.90
		Oil	9	36.25	2984.75
		Natural gas	0	2.085	2184.03
4	Retail and accommodation	Coal	12	3.75	1977.90
		Oil	9	5.77	2984.75
		Natural gas	0	1.462	2184.03
5	Consumption of living	Coal	12	1.88	1977.90
		Oil	9	16.7	2984.75
		Natural gas	0	0.736	2184.03

Based on Table 2, model 2.8, $C_{k}=(c_{ij}^{k}),k=1,2,3;i=1,2,\cdots ,5,j=1,2,3$ can be expressed as, where the superscript *k* denotes SO_2_, NO_X_, and CO_2_, *i* corresponds to the five different social sectors, and *j* corresponds to the three kinds of energies including coal, oil, and natural gas. For example, $c_{43}^{2}$ stands for the retail and accommodation sector coded by 4, consumes 1m^3^ natural gas, the energy is of the 3^rd^ type, and discharges 1.462x10^-3^ kg of NO_X_, the second kind of gas.

#### Constraint parameters in the optimization model 2.8

Based on the final consumptions of coal, crude oil products, and natural gas, the three major energy products by sectors in Tianjin in 2012 [21], and their coefficients of converting into standards, the final consumptions by each sector of Tianjin in 2012 and the final consumptions of converting into standard coal are obtained, as shown in Table 3.

**Table 3:** Final Consumptions of Energies in Tianjin.

Product No.	Main Industries	Standard coal *D*_*i*_(10^7^kg)	Final Consumptions		
			Coal (10^7^kg)	Oil (10^7^kg)	Natural gas (10^8^m^3^)
1	Large-scale agriculture	60.43	17.49	34.30	0
2	Industry	2029.72	970.08	791.85	16.25
3	Transportation	439.97	29.86	296.98	0.21
4	Retail and accommodation	166.97	32.50	43.23	5.83
5	Consumption of living	337.27	67.60	158.62	4.70
Total		2973.93	1117.5	1324.9	26.99

In this table, corresponding tothe energy demand constraints of model 2.8, *ρ*_*j*_ is the coefficient of converting the *j*th energy into standards, 0.71 for coal, 1.4 for oil products, and 14.3 for natural gas; and *D*_*i*_ is the energy demand of the *i*th sector, corresponding to the third column in the table.

The energy supply constraints of model 2.8 are further considered, as the three kinds of energies are limited in supply, and complementary on the premise of meeting the energy consumption. The supply of the *j*th energy product is 150% of the consumption of Tianjin in 2012 because of low value results in small adjustable space of the energy structure while high value results in the coal and oil are replaced by natural gas, which does not accord with the supply situation of natural gas.

### Solution analysis of the optimization model 2.8

Based on the actual data from Subsections 3.2.1–3.2.3, the reduction optimization model containing 15 non-negative decision variables, three linear objectives, and eight linear inequality constraints was set up. According to the introduction about instance data, after it is converted into the model of standard form, eight slack variables should be applied for it to become a multi-objective programming model containing 23 continuous variables, three linear objectives, eight linear equality constraints, and 23 inequality constraints.

Direct calculation involves $C_{28}^{3}=490314$ poles of the maximum and large amount of calculation. First, some problems can be simplified. If the converting coefficient *ρ* and emission coefficient *C* are non-negative, $\bar{x}$ is an efficient solution of the model, the target value $\bar{y}=\{{\bar{y}}_{1},{\bar{y}}_{2},{\bar{y}}_{3}\},$ and if *i*_0_ ∈ {1,2,3,4,5} makes $\rho_{1}\bar{x_{i1}}+\rho_{2}\bar{x_{i2}}+\rho_{3}\bar{x_{i3}}>D_{i}$, i.e., the total energy allocated to a sector exceeds the demand, xi1'<=xi1¯ xi2'<=xi2¯ xi3'<=xi3¯, and *ρ*_1_*x*_*i*1_' + *ρ*_2_*x*_*i*2_' + *ρ*_3_*x*_*i*3_' = *D*_*i*_, then a new solution is obtained.

x'=(xij)={xij=xij¯,i≠i0xij=xij',i=i0,xd=−Ax'+b

The new solution above is another efficient solution to the model, and its corresponding target value $y'\leq \bar{y}=\{{\bar{y}}_{1},{\bar{y}}_{2},{\bar{y}}_{3}\}.$ $\bar{y}$ is whereinis an efficient solution (i.e $\bar{y}\leq y'$, $\bar{y}=y'$). Therefore, if a solution is efficient, then the responding demand inequality degenerates into an equality constraint. Five slack variables can be removed from the model, eventually forming a multi-objective model with the maximum poles of $C_{18}^{8}=43758$. Notice that the pole number has been greatly reduced.

With the above model parameters and algorithm, MATLAB can be used in the programing. As this paper mainly studies about reasonable emission reduction scheme, similar emission schemes can be regarded as equivalent. To get the calculation speed, calculation precision is set as 3, and the solution steps are described as follows.

**Fig 2:**
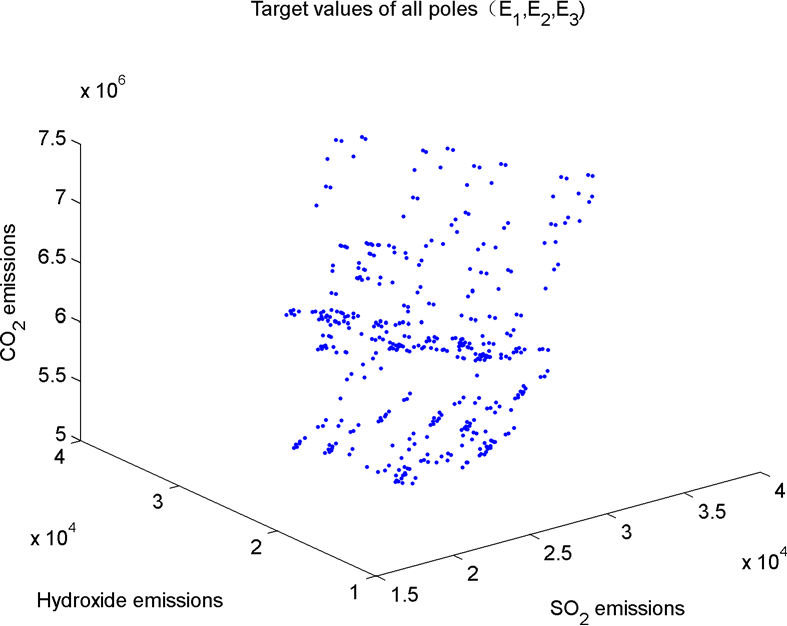
Target values of all poles.

First, get the emission values of the three kinds of gases in all poles of collaborative reduction, as shown in Fig 2.

**Fig 3:**
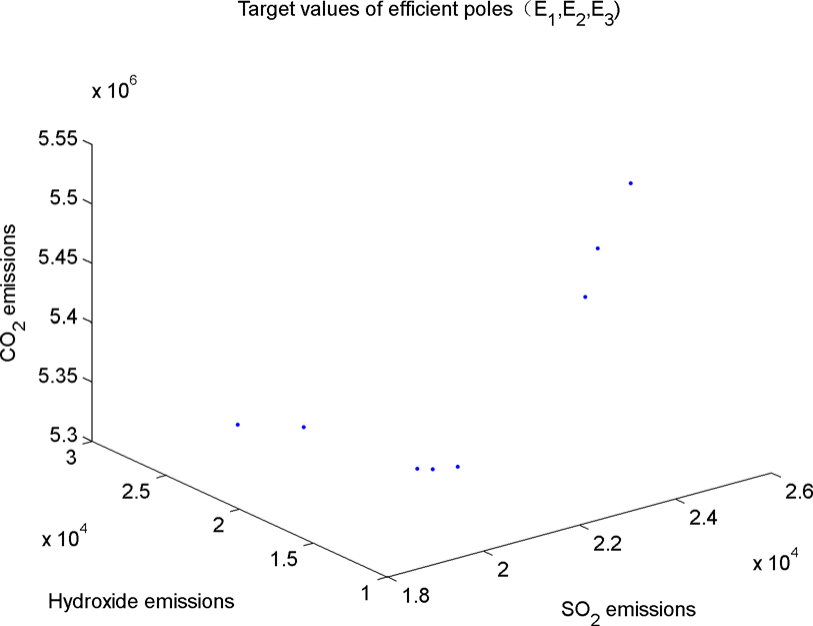
Target values of all efficient solutions.

Second, get the emission values of the three kinds of gases in all efficient poles of the model, as shown in Fig 3.

**Fig 4:**
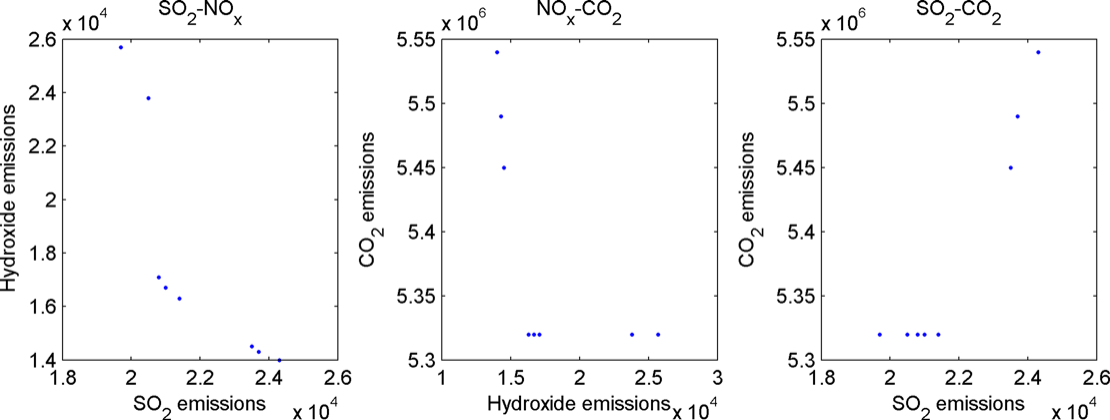
Pairwise comparisons of efficient solutions and target values.

Third, get the emission values of the three kinds of gases in all efficient poles. The pairwise comparisons are shown in Fig 4.

In Fig 4, the SO_2_ and CO_2_ of the efficient solution are positively correlated, i.e., when the SO_2_ emission of an efficient solution is lesser, the CO_2_ emission is also lesser correspondingly. Such correlation does not exist between SO_2_ and NO_X_.

**Fig 5:**
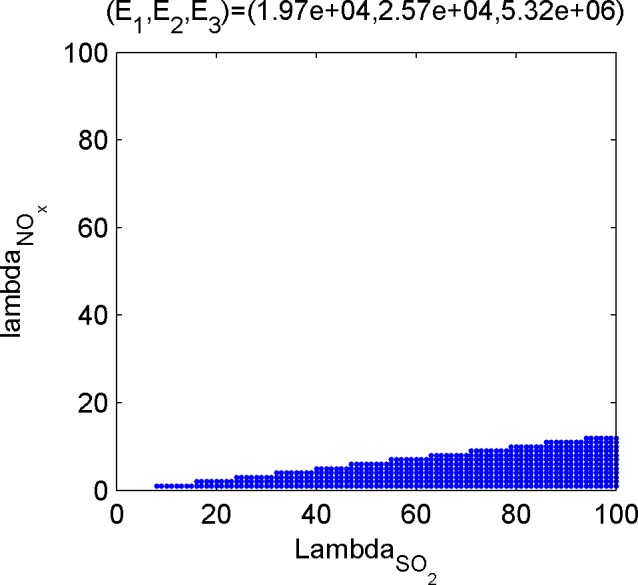
The dominant set relevant for (E_1_,E_2_,E_3_) = (1.97e+04,2.57e+04,5.32e+06).

**Fig 6:**
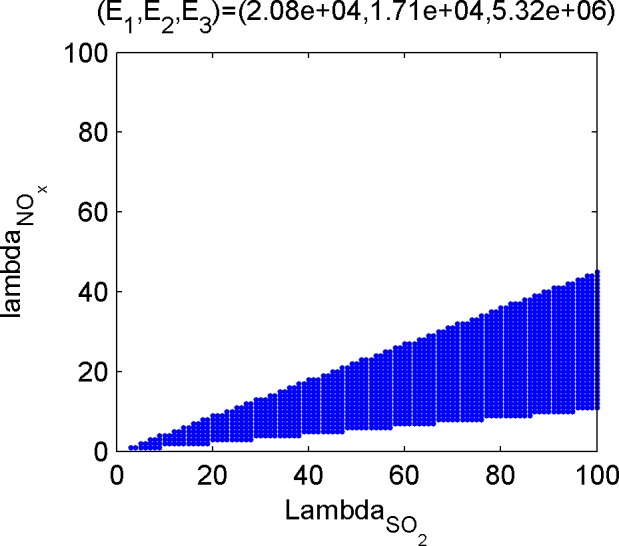
The dominant set relevant for (E_1_,E_2_,E_3_) = (2.08e+04,1.71e+04,5.32e+06).

**Fig 7:**
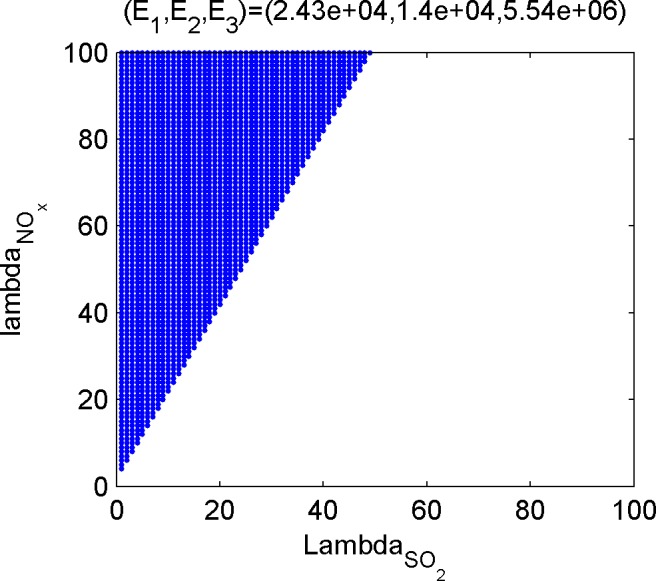
The dominant set relevant for (E_1_,E_2_,E_3_) = (2.43e+04,1.4e+04,5.54e+06).

**Fig 8:**
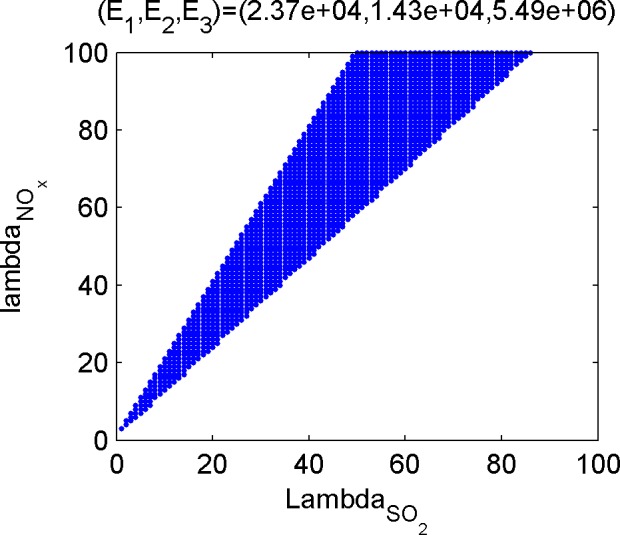
The dominant set relevant for (E_1_,E_2_,E_3_) = (2.37e+04,1.43e+04,5.49e+06).

Last, calculate the dominant set of the efficient poles. The dominant set is the set of emission weights of the three kinds of gases, and the subset in ${\mathbb{R}}_{++}^{3}$. For the convenience of discussion, the weight of CO_2_ is set to be 1, and the position of 2-D point composed of weights of the other two gases on the plane is used to describe the dominant set. Fig 5, Fig 6, Fig 7 and Fig 8 shows the dominant sets of efficient poles.

The dominant sets of efficient solutions divide the plane ${\mathbb{R}}_{++}^{2}$ into seven mutually disjoint parts, suggesting that different efficient solutions correspond to different preference information. Therefore, if the tendencies of the three kinds of pollutants are given, even if there is no accurate weight ratio, the optimal condition of the corresponding emissions can be achieved.

Based on the results of the optimization model in Section 3.3 and the efficient solutions under different preference information (Fig 5, Fig 6, Fig 7 and Fig 8), several kinds of special and important preference information are given to analyze the corresponding optimized emissions and the corresponding energy configuration suggestions, and the following suggestions are given for environmental protection and economic development in Tianjin.

#### Scheme focusing on SO_2_ emission reduction

If SO_2_ is taken as the main object in air pollutant control and its weight is higher than that of NO_X_, refer to Fig 5 or Fig 6, and place extra emphasis on Fig 5. According to the optimization scheme of Fig 6, SO_2_ and NO_X_ emissions are 208,000 and 171,000 tons, respectively. According to the energy configuration scheme, the consumption of natural gas should be equal to the amount of supply to achieve the optimum. In Fig 5 with higher weight of SO_2_, gas use in industrial sectors should be increased by more than 25% so that SO_2_ emissions will be reduced by 11,000 tons.

#### Scheme focusing on NO_X_ emission reduction

If NO_X_ is taken as the main object in air pollutant control and its weight is higher than that of SO_2_, refer to Fig 7 or Fig 6, and place extra emphasis on Fig 7. According to the optimization scheme in Fig 7, the SO_2_, NO_X_, and CO_2_ emissions are 243,000, 140,000, and 5,540 tons, respectively, while that in Fig 8 are 237,000, 143,000, and 54.9 million tons, respectively. These two kinds of situations are caused by high NO_X_ emission coefficient of life-use crude oil, thus to reduce the NO_x_ emission, life consumption of oil type energies should be reduced.

## Discussion and Prospect

This paper used multi-objective optimization to conduct mathematical modeling, theoretical analysis, and empirical research on emission reduction of sulfur dioxide, nitric oxides, and carbon dioxide, and acquired several comprehensive emission reduction schemes which place particular emphasis on different objectives. Ever since China issued measures on energy conservation, emission reduction, and total quantity control of pollutants such as sulfur dioxide, the government has been more motivated to solve problems such as managingair pollution, energy conservation, and emission reduction, and investments made by the government have been increasing consistently. These investments are mainly used for structural adjustment, technology improvement, and measures of fuel gas desulfurization and denitration to reduce the harm of industrial waste gas in the air quality to an extreme degree. Balancing the quantity of pollutants discharged and energy usage schemes in control investments under insufficient funds in order to reach the optimal emission reduction strategy is a problem demanding prompt discussion in the future. By implementing theemission reduction of sulfur dioxide, nitric oxide, and carbon dioxide, as well as accumulation of specific investment data, control investments can be incorporated into the model to solve the optimization model of emission reduction with investments considered.

## Supporting Information

S1 File Energy Balance Table by Region (Tianjin). (PDF)
