# Supplementary material for: Collaborative Emission Reduction Model Based on Multi-Objective Optimization for Greenhouse Gases and Air Pollutants
Source: PLoS One. 2016 Mar 24;11(3):e0152057. doi: 10.1371/journal.pone.0152057 (PMC4806840; doi:10.1371/journal.pone.0152057)
Supplement: S1 File — (PDF) [file pone.0152057.s001.pdf]

6-2 天津能源平衡表(实物量)-2013

项目	Item	煤合计 (万吨) Coal Total (10^4 tons)	原煤 (万吨) Raw Coal (10^4 tons)
**一.可供本地区消费的能源量**	**Total Primary Energy Supply**	**5278.67**	**4832.50**
1.一次能源生产量	Indigenous Production		
2.外省(区、市)调入量	Moving In from Other Provinces	5312.48	4871.23
3.进口量	Import	2383.07	2383.07
4.境内轮船和飞机在境外的加油量	Domestic Airplanes&Ships Refueling in Abroad		
5.本省(区、市)调出量(-)	Sending Out to Other Provinces(-)	-2266.12	-2266.12
6.出口量(-)	Export(-)	-116.90	-116.90
7.境外轮船和飞机在境内的加油量(-)	Oversea Airplanes&Ships Refueling in China(-)		
8.库存增(-)、减(+)量	Stock Change	-33.86	-38.78
**二.加工转换投入(-)产出(+)量**	**Input(-) & Output(+) of Transformation**	**-4058.22**	**-3707.67**
1.火力发电	Thermal Power	-2702.33	-2702.33
2.供热	Heating Supply	-1005.34	-1005.34
3.洗选煤	Coal Washing		
4.炼焦	Coking	-350.55	
5.炼油及煤制油	Petroleum Refineries		
#油品再投入量(-)	Petroleum Products Input (-)		
6.制气	Gas Works		
#焦炭再投入量(-)	Coke Input (-)		
7.天然气液化	Natural Gas Liquefaction		
8.煤制品加工	Briquettes		
9.回收能	Recovery of Energy		
**三.损失量**	**Loss**	**17.23**	**17.23**
**四.终端消费量**	**Total Final Consumption**	**1203.22**	**1107.60**
1.农、林、牧、渔、水利业	Agriculture, Forestry, Animal Husbandry, Fishery and Water Conservancy	19.60	19.60
2.工业	Industry	991.47	895.85
#用作原料、材料	Non-Energy Use	121.05	120.95
3.建筑业	Construction	16.21	16.21
4.交通运输、仓储和邮政业	Transport, Storage and Post	28.58	28.58
5.批发、零售业和住宿、餐饮业	Wholesale, Retail Trade and Hotel, Restaurants	12.63	12.63
6.其他	Others	70.43	70.43
7.生活消费	Residential Consumption	64.30	64.30
城镇	Urban	4.01	4.01
乡村	Rural	60.29	60.29
**五.平衡差额**	**Statistical Difference**		
**六.消费量合计**	**Total Energy Consumption**	**5278.67**	**4832.50**

?1994-2016 China Academic Journal Electronic Publishing House. All rights reserved. http://www.cnki.net

Energy Balance of Tianjin (Physical Quantity) -2013

洗精煤 (万吨) Cleaned Coal (10^4 tons)	其他洗煤 (万吨) Other Washed Coal (10^4 tons)	型煤 (万吨) Briquettes (10^4 tons)	煤矸石 (万吨) Gangue (10^4 tons)	焦炭 (万吨) Coke (10^4 tons)	焦炉煤气 (亿立方米) Coke Oven Gas (10^8 cu.m)	高炉煤气 (亿立方米) Blast Furnace Gas (10^8 cu.m)	转炉煤气 (亿立方米) Converter Gas (10^8 cu.m)	其他煤气 (亿立方米) Other Gas (10^8 cu.m)
**427.18**	**17.62**	**1.37**	**16.36**	**695.29**	**1.93**	**0.01**		
423.40	16.55	1.30	16.34	690.84	4.20	10.56	0.64	
				-7.71	-2.27	-10.55	-0.64	
3.78	1.07	0.07	0.02	12.16				
**-350.55**				**260.19**	**3.44**	**184.46**	**9.82**	
					-1.40	-14.89	-2.40	
-350.55				260.19	4.84			
						199.35	12.22	
**76.63**	**17.62**	**1.37**	**16.36**	**955.48**	**5.37**	**184.47**	**9.82**	
76.63	17.62	1.37	16.36	955.48	5.37	184.47	9.82	
		0.10	7.72	0.84				
**427.18**	**17.62**	**1.37**	**16.36**	**955.48**	**6.77**	**199.36**	**12.22**	

?1994-2016 China Academic Journal Electronic Publishing House. All rights reserved. http://www.cnki.net

6-2 续表 1

项 目	Item	其他焦化产品(万吨) Other Coking Products (10^4 tons)	油品合计(万吨) Petroleum Products Total (10^4 tons)
**一.可供本地区消费的能源量**	**Total Primary Energy Supply**	**51.69**	**1542.18**
1.一次能源生产量	Indigenous Production		3044.53
2.外省(区、市)调入量	Moving In from Other Provinces	68.28	3653.08
3.进口量	Import		1343.63
4.境内轮船和飞机在境外的加油量	Domestic Airplanes&Ships Refueling in Abroad		17.70
5.本省(区、市)调出量(-)	Sending Out to Other Provinces(-)	-16.92	-6462.02
6.出口量(-)	Export(-)		
7.境外轮船和飞机在境内的加油量(-)	Oversea Airplanes&Ships Refueling in China(-)		-67.08
8.库存增(-)、减(+)量	Stock Change	0.33	12.34
**二.加工转换投入(-)产出(+)量**	**Input(-) & Output(+) of Transformation**	**17.18**	**-133.05**
1.火力发电	Thermal Power		-24.43
2.供热	Heating Supply		-37.31
3.洗选煤	Coal Washing		
4.炼焦	Coking	17.18	
5.炼油及煤制油	Petroleum Refineries		272.42
#油品再投入量(-)	Petroleum Products Input (-)		-343.73
6.制气	Gas Works		
#焦炭再投入量(-)	Coke Input (-)		
7.天然气液化	Natural Gas Liquefaction		
8.煤制品加工	Briquettes		
9.回收能	Recovery of Energy		
**三.损失量**	**Loss**		**0.81**
**四.终端消费量**	**Total Final Consumption**	**68.87**	**1408.42**
1.农、林、牧、渔、水利业	Agriculture, Forestry, Animal Husbandry, Fishery and Water Conservancy		27.04
2.工业	Industry	68.87	818.79
#用作原料、材料	Non-Energy Use	44.99	214.52
3.建筑业	Construction		104.74
4.交通运输、仓储和邮政业	Transport, Storage and Post		234.99
5.批发、零售业和住宿、餐饮业	Wholesale, Retail Trade and Hotel, Restaurants		20.27
6.其他	Others		59.84
7.生活消费	Residential Consumption		142.75
城镇	Urban		127.94
乡村	Rural		14.81
**五.平衡差额**	**Statistical Difference**		**-0.10**
**六.消费量合计**	**Total Energy Consumption**	**68.87**	**1542.28**

?1994-2016 China Academic Journal Electronic Publishing House. All rights reserved. http://www.cnki.net

Continued 1

原油<br>(万吨)<br>Crude Oil<br>(10^4 tons)	汽油<br>(万吨)<br>Gasoline<br>(10^4 tons)	煤油<br>(万吨)<br>Kerosene<br>(10^4 tons)	柴油<br>(万吨)<br>Diesel Oil<br>(10^4 tons)	燃料油<br>(万吨)<br>Fuel Oil<br>(10^4 tons)	石脑油<br>(万吨)<br>Naphtha<br>(10^4 tons)	润滑油<br>(万吨)<br>Lubricants<br>(10^4 tons)	石蜡<br>(万吨)<br>Paraffin Waxes<br>(10^4 tons)	溶剂油<br>(万吨)<br>White Spirit<br>(10^4 tons)
**1759.15**	**0.93**	**-74.64**	**-334.88**	**68.82**	**72.84**	**29.74**	**0.17**	**0.93**
3044.53								
4.77	1292.08	15.11	2027.52	101.02	75.04	29.74	0.13	
1285.04		4.45	0.86	53.28				
		0.60		17.10				
-2598.82	-1289.38	-92.70	-2356.91	-32.63				
		-2.99	-0.90	-63.19				
23.63	-1.77	0.89	-5.45	-6.76	-2.20		0.04	0.93
**-1746.57**	**211.31**	**130.76**	**659.53**	**-19.88**	**173.20**			**3.52**
-7.90								
-1738.67	211.31	130.76	659.53	18.11	283.57			3.52
				-37.99	-110.37			
**0.81**								
**11.77**	**212.24**	**56.12**	**324.65**	**48.94**	**246.14**	**29.74**	**0.17**	**4.45**
	5.86		20.63			0.55		
11.77	11.96	0.75	48.45	1.44	246.14	29.19	0.17	4.45
0.12		0.24	1.10		184.78			
	13.90	0.07	86.75	3.62				
	30.94	54.11	107.24	42.61				
	7.50	0.91	10.88	0.21				
	17.15	0.28	40.84	1.06				
	124.93		9.86					
	118.06		8.83					
	6.87		1.03					
**0.00**					**-0.10**			
**1759.15**	**212.24**	**56.12**	**324.65**	**86.93**	**356.51**	**29.74**	**0.17**	**4.45**

?1994-2016 China Academic Journal Electronic Publishing House. All rights reserved. http://www.cnki.net

6-2 续表 2

项　　目	Item	石油沥青（万吨） Bitumen Asphalt (10^4 tons)	石油焦（万吨） Petroleum Coke (10^4 tons)
**一.可供本地区消费的能源量**	**Total Primary Energy Supply**	**0.19**	**-20.32**
1.一次能源生产量	Indigenous Production		
2.外省(区、市)调入量	Moving In from Other Provinces	0.19	3.95
3.进口量	Import		
4.境内轮船和飞机在境外的加油量	Domestic Airplanes&Ships Refueling in Abroad		
5.本省(区、市)调出量(-)	Sending Out to Other Provinces(-)		-22.76
6.出口量(-)	Export(-)		
7.境外轮船和飞机在境内的加油量(-)	Oversea Airplanes&Ships Refueling in China(-)		
8.库存增(-)、减(+)量	Stock Change		-1.51
**二.加工转换投入(-)产出(+)量**	**Input(-) & Output(+) of Transformation**		**95.19**
1.火力发电	Thermal Power		-16.51
2.供热	Heating Supply		-37.30
3.洗选煤	Coal Washing		
4.炼焦	Coking		
5.炼油及煤制油	Petroleum Refineries		149.00
#油品再投入量(-)	Petroleum Products Input (-)		
6.制气	Gas Works		
#焦炭再投入量(-)	Coke Input (-)		
7.天然气液化	Natural Gas Liquefaction		
8.煤制品加工	Briquettes		
9.回收能	Recovery of Energy		
**三.损失量**	**Loss**		
**四.终端消费量**	**Total Final Consumption**	**0.19**	**74.87**
1.农、林、牧、渔、水利业	Agriculture, Forestry, Animal Husbandry, Fishery and Water Conservancy		
2.工业	Industry	0.19	74.87
#用作原料、材料	Non-Energy Use		
3.建筑业	Construction		
4.交通运输、仓储和邮政业	Transport, Storage and Post		
5.批发、零售业和住宿、餐饮业	Wholesale, Retail Trade and Hotel, Restaurants		
6.其他	Others		
7.生活消费	Residential Consumption		
城镇	Urban		
乡村	Rural		
**五.平衡差额**	**Statistical Difference**		
**六.消费量合计**	**Total Energy Consumption**	**0.19**	**128.68**

?1994-2016 China Academic Journal Electronic Publishing House. All rights reserved. http://www.cnki.net

Continued 2

液化石油气<br>(万吨)<br>LPG<br>(10^4 tons)	炼厂干气<br>(万吨)<br>Refinery Gas<br>(10^4 tons)	其他石油制品<br>(万吨)<br>Other Petroleum Products<br>(10^4 tons)	天然气<br>(亿立方米)<br>Natural Gas<br>(10^8 cu.m)	液化天然气<br>(万吨)<br>LNG<br>(10^4 tons)	热力<br>(万百万千焦)<br>Heat<br>(10^{10} kJ)	电力<br>(亿千瓦小时)<br>Electricity<br>(10^8 kW•h)	其他能源<br>(万吨标煤)<br>Other Energy<br>(10^4 tce)
**-34.99**		**74.24**	**37.52**	**1.97**		**175.43**	**77.56**
			18.73			5.77	17.72
0.53		103.00	31.07	1.97		170.39	60.00
-39.70		-29.12	-12.28			-0.73	
4.18		0.36					-0.16
**81.77**	**60.51**	**217.61**	**-3.79**	**1.83**	**19407.75**	**619.05**	**-21.01**
-0.02			-0.51		-193.06	619.05	-10.96
	-0.01		-0.64		19059.00		-10.05
83.36	60.52	411.41	-2.35				
-1.57		-193.80					
			-0.29	1.83			
					541.81		
			**1.37**		**47.00**	**47.33**	
**46.78**	**60.51**	**291.85**	**32.36**	**3.80**	**19360.75**	**747.15**	**56.55**
						14.58	
37.05	60.51	291.85	18.86	1.97	9322.85	519.03	56.55
27.34		0.94	1.18	0.05			
0.40			0.03		76.97	14.43	
0.09			1.15	1.83	132.62	22.77	
0.77			6.18		347.14	35.27	
0.51			1.25		1119.36	65.90	
7.96			4.89		8361.81	75.17	
1.05			4.89		8361.81	55.40	
6.91						19.77	
**48.37**	**60.52**	**485.65**	**37.27**	**3.80**	**19600.81**	**794.48**	**77.56**

?1994-2016 China Academic Journal Electronic Publishing House. All rights reserved. http://www.cnki.net
